# Pros and Cons of (NH_4_)_2_S Solution Treatment of p-GaN/Metallization Interface: Perspectives for Laser Diode

**DOI:** 10.3390/ma17184520

**Published:** 2024-09-14

**Authors:** Iryna Levchenko, Serhii Kryvyi, Eliana Kamińska, Julita Smalc-Koziorowska, Szymon Grzanka, Jacek Kacperski, Grzegorz Nowak, Sławomir Kret, Łucja Marona, Piotr Perlin

**Affiliations:** 1 Institute of High Pressures Physics, Polish Academy of Science, 29/37 Sokołowska St., 01-142 Warsaw, Poland; julita.smalc.koziorowska@unipress.waw.pl (J.S.-K.); szgrzanka@unipress.waw.pl (S.G.); jacek@unipress.waw.pl (J.K.); grzegorz.nowak@unipress.waw.pl (G.N.); lucja@unipress.waw.pl (Ł.M.); piotr@unipress.waw.pl (P.P.); 2 Institute of Physics, Polish Academy of Sciences, 32/46 Al. Lotników, 02-668 Warsaw, Poland; kryvyi@ifpan.edu.pl (S.K.); kret@ifpan.edu.pl (S.K.); 3 Institute for Nanoscience and Engineering, University of Arkansas, 731 W Dickson St Suite 103, Fayetteville, AR 72701, USA

**Keywords:** gallium nitride (GaN), wet treatment, sulfur-based solution, interface, diffusion, ohmic contact, specific contact resistance (*ρ_c_*), solid solution

## Abstract

The impact of wet treatment using an (NH_4_)_2_S-alcohol solution on the interface state of the p-GaN/Ni/Au/Pt contact system and laser diode processing was investigated. Sulfur wet cleaning resulted in reduced surface roughness and contact resistivity. The lowest specific contact resistance (*ρ_c_* < 1 × 10^−4^ Ω·cm^2^) was achieved with samples treated with an (NH_4_)_2_S-isopropanol solution, whereas the highest resistivity (*ρ_c_* = 3.3 × 10^−4^ Ω·cm^2^) and surface roughness (*R_a_* = 16 nm) were observed in samples prepared by standard methods. Annealing the contact system in an N_2_ + O_2_ + H_2_O atmosphere caused degradation through species inter-diffusion and metal-metal solid solution formation, irrespective of the preparation method. Standard prepared substrates developed a thin GaN-Au intermediate layer at the interface after heat treatment. Enhanced adhesion and the absence of GaN decomposition were observed in samples additionally cleaned with the (NH_4_)_2_S-solvent solution. Complete oxidation of nickel to NiO was observed in samples that underwent additional sulfur solution treatment. The intensity of metal species mixing and nickel oxidation was influenced by the metal diffusion rate and was affected by the initial state of the GaN substrate obtained through different wet treatment methods.

## 1. Introduction

Semiconductor surface properties are constrained by the near-surface layer’s structure, morphology, chemical composition, sensitivity, and stability. Due to surface reconstruction effects, the morphology and electrical characteristics of a semiconductor surface differ significantly from those of the bulk material, driven by a reduction in substrate surface energy [1].

III-V semiconductor surfaces are known to be affected by a high density of surface states [2]. These states often arise from extrinsic effects such as contaminations or defects on the substrate surface. For example, contaminations can create adsorption-induced surface states.

There are two primary sources of surface states within the band gap of III-V semiconductors [3]. The first source is local strain at the contamination/semiconductor interface. The second source is defects caused by deficiencies in group III and group V elements, such as antisites or vacancies. These defects result in a high density of midgap surface states (due to V deficits or antisites) and a low density of surface states near the valence band (due to III deficits or antisites). To mitigate the influence of surface states, it is essential to remove impurities and oxides or deactivate antisite and element deficit defects.

In the case of GaN (0001), two types of surface state densities are observed. The higher density states are unoccupied and primarily originate from gallium (Ga) dangling bonds. The lower density states are occupied and are localized on bonds between a gallium adatom and three gallium surface atoms [4]. Most semiconductor defects occur in the near-surface region within 1 nm, which is considered the active region of fabricated devices. Mitigating these surface states is crucial for improving the performance and reliability of semiconductor devices.

Antisite defects on III-V semiconductors contribute to poor electrical properties of the surface [3]. The interaction between foreign elements and the semiconductor substrate leads to the formation of a disordered crystal structure and increases the density of surface states. By eliminating dangling bonds or impurity atoms, recombination centers caused by various surface states can be terminated [5]. The oxidized phases of III/V group elements induce concentration-dependent, oxygen-induced structural disorder that results in gap states [6].

The Ga-polar surface of GaN is highly sensitive to chemisorption of oxygen and water at room temperature, leading to surface passivation, oxidation, and increased ohmic contact resistivity [7]. The native oxides of III-V semiconductors are complex, are highly defective due to chemical inhomogeneity, and typically exhibit an amorphous structure [6]. Surface oxides can act as barriers to carrier transport from metal to semiconductor, thereby increasing ohmic contact resistivity. It can be reduced by oxide chemical removing before metal deposition.

Chemisorbed contaminants on a clean GaN (0001) surface can exhibit different properties compared to atoms incorporated into the semiconductor crystal lattice. The semiconductor surface is also influenced by adsorbate-induced surface dipoles [8]. In GaN-based light emitting diodes (LEDs), surface dislocations can facilitate metal migration, leading to increased leakage current and potential device failure [9]. Negatively charged threading dislocations in GaN substrate do not create localized current leakage paths. Instead, pure edge and mixed dislocations are negatively charged and exhibit low conductivity, whereas pure screw dislocations cause localized leakage paths and appear to lack measurable charge [10]. Therefore, minimizing interface states and the defect-free semiconductor substrate are crucial for optimal device performance.

Various methods of GaN surface modification, including physical adsorption and covalent bond formation, aim to reduce the surface work function of the semiconductor. These modifications enhance the performance and longevity of electronic and optoelectronic devices. Surface functionalization of III-V semiconductors can be achieved using organic molecules (such as thiols and phosphoric acid) or inorganic materials (such as oxides, SiN, and chalcogenides) [11]. The ionic nature of the chemical bonds at the wafer surface and the trapping of carriers by surface traps can influence the dipole moment, modify the electric field, and lead to charge redistribution on the surface.

The Ga-face of GaN exhibits lower electron affinity compared to the N-face, resulting in higher Schottky barrier heights: approximately 1 eV for n-type substrate material and about 2 eV for p-type materials [12,13]. The barrier height is influenced by interface states, which arise from surface states, metal-induced gap states, and chemical reactions at the metal-semiconductor interface. Reducing the barrier height cannot be achieved by merely selecting different metals; instead, introducing impurities on the semiconductor surface to create surface states can pin the Fermi level [14]. The processes existing on the surface are attributed to the generation of thermodynamically stable states. When metals are deposited onto a GaN substrate, a high-resistance rectifying Schottky barrier forms due to the significant mismatch between metal and semiconductor Fermi levels [15].

Thermal treatment was recommended as an effective final step of GaN cleaning. However, applied high temperature is not acceptable for the laser diode (LD) fabrication process due to the thermal degradation. Moreover, such treatment can disrupt the metal-semiconductor structure and deteriorate the electrical properties of the samples. This effect is due to mechanical stress from differing thermal expansion coefficients between the film and substrate and the low adhesion energy of the binding [16].

Contact annealing often involves solid-phase reactions and the incorporation of secondary passivating species like hydrogen, which can interact with native defects, impurities, and dangling bonds in the semiconductor. In p-type semiconductors, hydrogen atoms form donor-like species (H^+^), which coulombically attract acceptors [17]. Heavily Mg-doping GaN substrates to achieve ohmic contacts is often eliminated by self-compensation effects and the inhomogeneous distribution of dopants.

Chemical procedures such as cleaning, etching, and passivation have distinct effects on the surface band bending of semiconductors. The initial preparation of the semiconductor surface strongly influences the metal/GaN interface morphology and contact I-V characteristics. Channel mobility, for instance, depends on scattering at the interface due to charges [18]. Current drift arises from electron trapping at interface states. Minimizing the resistance of ohmic contacts is crucial to reduce the device’s specific on-resistance and power losses.

Preparing a surface state with avoidance stoichiometric deficits is essential in the LD fabrication process. Dry cleaning may introduce surface disorder and damage, rendering the substrate electrically unsuitable [19]. Significant attention has been directed towards wet etching of III-V semiconductors using chalcogen-containing solutions, especially sulfur-based ones (e.g., P_2_S_5_/NH_4_OH, Na_2_S, SeS_2_ and (NH_4_)_2_S, CH_3_CSNH_2_) [20]. These etchants effectively remove native oxides by bonding with chalcogen atoms, thereby eliminating undesirable surface states [18].

Sulfur-based solutions target electrically active surface defects [21]. The interaction mechanism between sulfur and the semiconductor is described elsewhere [2]. The further reactivity will be predominantly eliminated by the formation of the Ga_x_S_y_ film on the surface [22]. The passivation effect depends on the breaking of III-V bonds and the formation of sulfur-semiconductor bonds. Ga-S bonds remain stable at temperatures less than 500 °C [22,23]. The stability of III-S bonds is due to the presence of 2.0 electrons and unfilled antibonding levels, unlike V-S bonds, which contain 2.25 electrons [3,24]. The excess 0.25 electrons in the antibonding level weakens B^V^-S bonds. Also, surface stabilization through the formation of A^III^-chalcogen bonds and electron transfer to dangling bonds has been reported [25].

It should be noted that the formation of A^III^-S or B^V^-S covalent bonds largely depends on the presence of the elemental form of the semiconductor component [26]. Thus, the initial state of the semiconductor surface can be modified according to the required cleaning or passivation effect.

From a surface stability perspective, treatment with (NH_4_)_2_S solution is preferred over Na_2_S-based treatments. This preference is due to the formation of stronger A^III^-S bonds and the absence of extra surface states. Cleaning with (NH_4_)_2_S solution maintains the stoichiometric etching of the semiconductor, decreases defective interfacial states of metal/semiconductor contacts, and stabilizes the GaN surface density of states [25,27]. The ammonium ion (NH_4_^+^) is highly volatile, making it a more favorable non-metallic component compared to Na_2_S. After treatment, (NH_4_)_2_S desorbs cleanly from the surface [28], whereas Na_2_S tends to leave behind undesirable sodium ions and exhibits a non-uniform etch rate [29]. Additionally, Na_2_S has a stronger passivating effect [30,31], which, in the case of laser diode (LD) fabrication, can lead to weaker bonding between the semiconductor and metals. Treatment with (NH_4_)_2_S solution enhances electron transfer from the semiconductor to the surface layer. It also prevents the formation of nanocrystals caused by GaN-metal lattice mismatch [32].

Alcoholic sulfur-based solutions are considered more effective than aqueous solutions. They increase the total amount of sulfides and provide stronger protection against wafer oxidation. In alcohol, solvated sulfide ions become more nucleophilic and have higher global hardness. This enables them to donate electrons and form strong covalent bonds with surface gallium atoms. However, sulfide ions in the first place prefer to passivate the dangling bonds of surface B^V^ atoms [20]. Only after that do they attack gallium atoms.

It should be emphasized that the generation of sulfur phases can be energetically unfavorable if antibonding states are filled [33]. Additionally, the insulating behavior of GaS is undesirable for LD processing. Despite these issues, sulfur-based solutions have potential as effective non-aggressive reagents for semiconductor cleaning (removing of an insulating layer) and modification of atomic arrangement on the surface.

Experimental data show that ammonium sulfide passivation depends on treatment time, solution temperature, and composition [34,35]. (NH_4_)_2_S-based etchant can be used for chemical and/or electrical passivation by neutralizing the dangling bonds as well as forming of the protective layer on the semiconductor surface. It should be noted that sulfur-based etchants are characterized by a slow etching rate [36]. Given that, short-term etching primarily cleans the surface rather than causing semiconductor dissolution or passivation.

This paper investigates the influence of sulfur-based solutions on the state of the p-GaN/metallization interface and correlates these effects with contact morphology and electrical properties from the point of view of the LD fabrication process.

## 2. Materials and Methods

### 2.1. Experimental Procedure

All experiments were conducted on GaN/Al_2_O_3_ templates with the structure design corresponding to that typical for nitride-based typical LD configurations. Samples were grown using metalorganic vapor phase epitaxy (MOVPE, Aixtron CSS 3 × 2″, Cambridge, UK). These structures comprised a 2 µm GaN layer, followed by a 500 nm Mg-doped GaN layer (with a free hole concentration of 5 × 10^17^ cm^−3^), and a 10 nm GaN-p++ sub-contact layer featuring an Mg concentration of 2 × 10^20^ cm^−3^.

Prior to metal contact deposition, the surface of the p-GaN samples underwent cleaning as previously described [37]. An additional short-time etching step using an (NH_4_)_2_S-solvent solution was performed before metal deposition. To prevent passivation film deposition on the wafer surface, the solution was stirred at 200 rpm. Isopropanol (CH_3_)_2_CHOH), t-butanol (t-(CH_3_)_3_COH), and water (H_2_O) were used as solvents.

Following the chemical treatment, Ni/Au/Pt metallization composition was deposited using an e-beam evaporator at a pressure of 2 × 10^−7^ Torr. Thermal annealing was conducted in a furnace at 500 °C in an oxidizing atmosphere (N_2_ + O_2_ + H_2_O) for 30 min.

### 2.2. Characterization Methods

Several characterization methods were employed to assess the properties of the semiconductor surface, metal contacts, and p-GaN/metallization interface, as follows:

- Transmission Line Method (TLM): used to ascertain the specific contact resistance by analyzing the I–V characteristics.
- Scanning Electron Microscopy (SEM): conducted with a Zeiss microscope (Carl Zeiss NTS Ltd., Oberkochen, Germany) at 4 kV to visualize the surface morphology of the etched semiconductor and annealed metallization.
- Atomic Force Microscopy (AFM): performed with a Dimension Nanoscope IV (Digital Instruments, currently Brucker, Tonawanda, NY, USA) to examine surface topography.
- Transmission Electron Microscopy (TEM): used to observe alterations in the metallization and GaN structures post-treatment with (NH_4_)_2_S-based solution.
- Energy Dispersive X-ray Spectroscopy (EDX): provided insights into the contact composition, metal distribution, and interface state of the GaN/Ni/Au/Pt contact system.
- Conductive Atomic Force Microscopy (c-AFM): the conductivity of the substrate surface was measured with Ntegra microscope (NTMdt, Moscow, Russia) under air ambient conditions.

### 2.3. Detailed Analytical Techniques

TEM (FEI, USA) and HR-TEM (E.A. Fischione Instrumental Inc., Export, PA, USA): structural examinations and elemental composition evaluations were conducted using an FEI Titan Cubed 80–300 microscope operating at 300 kV with objective lens correction. HR-TEM images were captured using a Gatan Ultrascan 1000 CCD camera (GATAN, Pleasanton, CA, USA).

HR-STEM: high-resolution scanning transmission electron microscopy in annular dark field mode was performed using a Fischione 3000 detector (E.A. Fischione Instrumental Inc., Export, PA, USA):. High-resolution images were acquired for GaN [11$\bar{2}$0] zone axis (ZA) orientation (electron beam is parallel to [11$\bar{2}$0] crystallographic direction).

FFT Analysis: fast Fourier Transformation (FFT) of HR-TEM and HR-STEM images was used to define the crystallographic orientation of metal layers relative to the GaN substrate. FFT in each case was scaled according to the theoretical inter-planar values of GaN.

EDX Mapping: collected in TEM using an EDAX 30 mm^2^ Si(Li) detector with a collection angle of 0.13 srad (EDAX of Ametek, Inc./GATAN, Pleasanton, CA, USA). The integration time for EDX acquisition was set at 1 s per pixel.

FIB Preparation: thin lamellas for TEM examination were prepared using the focused ion beam technique in the Helios Nanolab 600 (Thermo Fisher Scientific Inc., Waltham, MA, USA). A platinum protection layer was deposited to safeguard the top surface, and the thin lamellas were transferred onto a copper TEM grid.

c-AFM studies: the distribution of the surface current flowing through the junction and the topography were recorded simultaneously in contact mode. Voltages of +1 V were applied to the contact system, both in the standard-cleaned state and after additional etching in (NH_4_)_2_S solutions. The current through the tip was measured simultaneously with topography and recorded as a function of tip position.

## 3. Results and Discussion

Generally, GaN demonstrates excellent chemical resistance, being substantially insoluble in water, acid, or alkali solution under room conditions [38]. Chemical reactions between GaN and (NH_4_)_2_S are unlikely unless sulfur species are activated or the GaN substrate has defects providing a low energy path. Therefore, etching of epitaxially grown p-GaN by (NH_4_)_2_S-based solutions is likely to be dominated by physical or chemical adsorption rather than chemical reactions. Only excited sulfur atoms may break Ga-N bonds on an ordered, clean surface, forming covalent bonds with gallium atoms. This is consistent with the formation of strong A^III^-S and B^V^-S bonds observed in ultra-high-vacuum annealing processes, although such conditions are not used in our LD fabrication technology.

### 3.1. Morphology State

**Figure 1:**
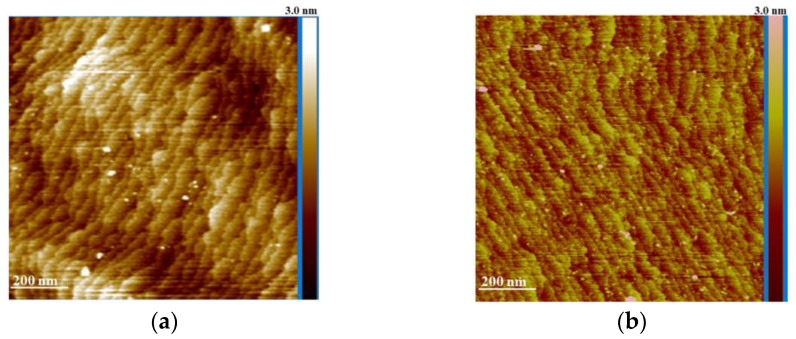
AFM overview scan of p-GaN surface after (**a**) standard cleaning and (**b**) standard and (NH_4_)_2_S-t-(CH_3_)_3_COH treatment.

The effect of GaN cleaning with the standard procedure and additional treatment in the (NH_4_)_2_S-based solution is shown in Figure 1. Atomic traces are present in both cleaning scenarios. The standard cleaning procedure affects the GaN surface but does not significantly alter the visibility or arrangement of the atomic-level traces. The (NH_4_)_2_S-based solution appears to act as a superior cleaning agent compared to the standard procedure. Additional cleaning in the (NH_4_)_2_S-solvent solution smooths the initial surface GaN morphology, resulting in a more visible and possibly more ordered near-surface structure.

The increase in visibility of atomic traces post-treatment with the (NH_4_)_2_S-based solution suggests that the solution may remove a layer of contaminants or oxides that obscure these traces. However, the fact that atomic traces are observed in both cleaning methods indicates that the underlying atomic structure of GaN is robust and persists through various cleaning procedures. Improvement of surface morphology is crucial for LD performance, which requires high precision and minimal surface defects.

A 30-min treatment with (NH_4_)_2_ solution revealed 2–4% sulfur film on the InGaN surface [39]. Sulfur film deposition follows with the gradual and somewhat inhomogeneous passivation process. Typically, when a passivation film forms on the semiconductor surface, it prevents further material removal and affects process uniformity. Prolongation of the treatment time up to 30 min reduced InGaN surface roughness from 4 nm to less than 1 nm.

In our experiments, the initial surface roughness (R_a_) after standard cleaning was 0.29 nm. Additional treatment with (NH_4_)_2_S solution resulted in a slight improvement in surface topography, reducing R_a_ to 0.25 nm. Comparing our AFM data with those represented in [39,40], it is clear that a more pronounced reduction in surface roughness occurs on irregular surfaces with a higher concentration of active sites. The smaller change observed in our experiments can be attributed to the relatively good initial condition of the surface. Nonetheless, even minor modifications in surface topography can significantly affect the morphology of the fabricated contact, as will be discussed later.

Since we used a shorter treatment time in our experiments, we believe these conditions were sufficient to achieve surface cleaning while avoiding the formation of a sulfur layer. Obtained data suggest that (NH_4_)_2_S-based solutions can effectively smooth semiconductor surfaces, with the outcome dependent on the initial surface condition and treatment duration. Depending on the desired final effect, sulfur solutions can be employed for either cleaning (short treatment) or passivation (extended treatment).

Comparing different (NH_4_)_2_S-solvent compositions, treatment with the (NH_4_)_2_S-t-(CH_3_)_3_COH solution shows improvement in the adhesion of the GaN surface. It reduces the interface trap density at the metal-semiconductor interface. Consequently, better fitting between the semiconductor and metal can be achieved.

**Figure 2:**
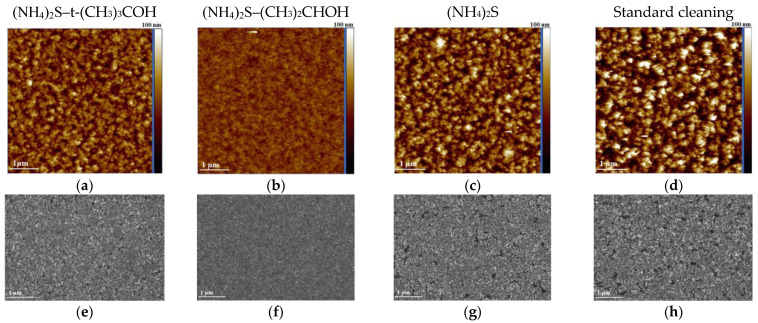
AFM maps (**a**–**d**) and SEM images (**e**–**h**) of the surface of annealed p-GaN/Ni/Au/Pt contact system.

Additionally, AFM measurements of samples prepared with different compositions were carried out after metallization deposition and contact annealing. Obtained data emphasize a uniform grainy structure at the nanometer scale, indicating a more controlled and homogeneous formation of the metal contact layer (Figure 2a–d). The reduction in grain size with additional (NH_4_)_2_S treatment suggests that the solution helps in refining the microstructure of the metal contacts, leading to more consistent and potentially superior electrical properties. Sample morphology with standard wet preparation exhibits the most non-uniform microstructure.

The roughness (R_a_) of the annealed metallization on the GaN substrate varies significantly, from approximately 4.0 nm to 16.0 nm, depending on the solvent used. Comparing with initial substrate surface roughness, it should be noted that even a negligible difference in the initial roughness of the semiconductor surface provides a valuable effect on the morphology of the annealed metallization. The (NH_4_)_2_S-isopropanol solution led to the formation of grain clusters with the lowest observed roughness. Reducing surface roughness may improve the semiconductor surface structure, thereby decreasing surface recombination.

According to SEM data, treatment with a sulfur-based solution reduces gaps in the metallization and promotes a tighter fit of the annealed composition (Figure 2e–g). This tighter fit can enhance electrical conductivity and mechanical stability of the metallization.

The hillock morphology of the metallization may be associated with the activation of metal mobility during annealing, differences in metal surface stress, and the formation of agglomerates on the surface [41]. The irregular holes visible in Figure 2e,g,h appear to distribute randomly and are not linked to individual dislocations, suggesting other factors, such as inconsistencies in the deposition process or localized stress variations. Heterogeneity in the shape of the annealed metallization is also expected due to differences in atomic radius (R(Ni) = 1.245 Å, R(Au) = 1.44 Å) and surface energy (E(Ni) = 149 meVÅ^−2^, E(Au) = 96.8 meVÅ^−2^) [42]. These properties have a direct influence on atom interaction and diffusion during the annealing process.

A more uniform and finer grain structure in the metal contacts can lead to more reliable and stable electrical characteristics, essential for the consistent operation and performance of LDs. Incorporating a sulfur-based solution into the cleaning and preparation process can optimize the fabrication process of GaN-based devices, ensuring better adhesion, lower interface trap densities, and improved surface uniformity.

### 3.2. Electrical Properties

**Figure 3:**
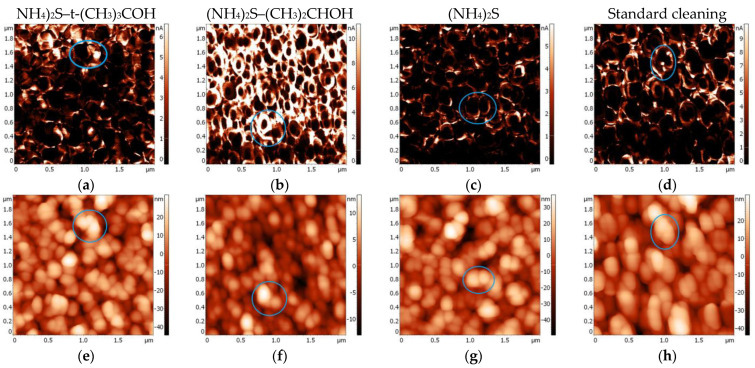
c-AFM current maps (**a**–**d**) and AFM images (**e**–**h**) of annealed p-GaN/Ni/Au/Pt.

Effective surface treatments that reduce the number of electrically active interface states can result in lower leakage currents and higher carrier mobility. The influence of wet chemical treatment on the contact surface potential has been investigated with local current measurements by the c-AFM method. The current maps allowed us to visualize the nanoscopic current flow through the contact area and establish a correlation with the annealed contact microstructure. Figure 3a–d illustrates electrically active interface states that were observed at an applied voltage of one volt in forward bias condition. Appropriate AFM data of surface morphology are shown in Figure 3e–h. Blue circles indicate the same place on current maps and the corresponding surface morphology.

Samples treated with the sulfur solution exhibit larger current areas in comparison with standard prepared sample. The reacted layer contains phases with localized regions displaying higher current levels and are arranged differently in contact structure. Grains with different conductive phases are randomly distributed. Obtained data demonstrate that the grains provide preferential paths for the current conduction through the contact. Detectable currents are predominantly observed at the edges of the grains, which act as charge-carrier transport channels. The midpoints of the metallization grains, conversely, show the lowest carrier flow. The uneven current flow is probably attributed to differences in the diameters of metallization grains. Larger grains at the edges enhance local conductivity, whereas smaller grains or inconsistencies in grain size can disrupt uniform current flow.

After annealing, the metallization arrangement reproduces the semiconductor surface morphology (Figure 3e–h). Surface analysis also suggests that the higher roughness of contacts tends to be responsible for the dropping in current. Surface shaping hinders carrier transport, likely due to limitations imposed by the density of states or the lifetime for charge transfer.

The variation of local conductivity can be associated with thickness differences of the metallization [41]. Moreover, areas with less conductivity may suggest the occurrence of charge trapping effects [43]. Dependencies of surface morphology-current areas highlight the effectiveness of the (NH_4_)_2_S-solvent treatment in reducing charge accumulation and improving conductive behavior.

The correlation between treatment composition, contact surface roughness, and resistivity (measured with TLM) is represented in Table 1.

The characteristics of annealed contacts significantly change with different chemical treatments. For the (NH_4_)_2_S-treated samples, the specific contact resistance (*ρ_c_*) is reduced to less than 1 × 10^−4^ Ω·cm^2^. The sample cleaned with (NH_4_)_2_S-isopropanol exhibits the lowest surface roughness and specific contact resistance. In contrast, the highest *ρ_c_* value, approximately 3.3 × 10^−4^ Ω·cm^2^, corresponds to the substrate with standard preparation. The higher resistivity in samples without sulfur-based treatment may be attributed to interface degradation and structural discontinuities such as island formation, which hinder efficient charge transport [44]. The reduction in *ρ_c_* value of the annealed Ni/Au/Pt contact can be attributed to an increase in hole concentration in the p-GaN epilayer [45]. This enhancement in carrier concentration may improve the overall conductivity of the contact.

According to the literature, pure and aqueous (NH_4_)_2_S solutions exhibit weaker chemical activity compared to alcohol-containing solutions. This is attributed to the enhanced electrostatic interaction between sulfur ions and the semiconductor surface atoms in the latter [46]. Our AFM data showing changes in surface roughness support this observation. However, in terms of contact resistance, there are discrepancies when comparing the effects of (NH_4_)_2_S treatment with surface morphology. We suspect that, in this case, the data may be misrepresented due to increased measurement error, which tends to rise with surface roughness. Nonetheless, the general trend of surface roughness correlating with contact resistance remains consistent.

According to [3], the reactivity of sulfur in solution strongly depends on the solvent’s dielectric constant (ε). t-butanol, with ε = 12.47, should enhance sulfur reactivity more than isopropanol, which has a dielectric constant of ε = 20.18. However, our data suggest that treatment with (NH_4_)_2_S-isopropanol may not be the sole factor influencing the effectiveness of the (NH_4_)_2_S-based solution in smoothing the substrate surface and reducing specific contact resistance. No clear dependency on the solvent nature was observed in our experiments. Achieved data support the idea that alcohol solvents with higher dielectric constants have less influence on (NH_4_)_2_S-based solution compared to Na_2_S-ines [20]. This discrepancy could be stimulated by the higher sensitivity of t-butanol to temperature conditions or the weak stability of the solution during heating. Such behavior makes composition control challenging and could diminish the expected benefits of using t-butanol as a solvent.

Principally, (NH_4_)_2_S may dissociate in several redox reactions [47]:

(NH_4_)_2_S → 2NH_4_^+^ + S^2−^ (1)

S^2−^ + H_2_O ↔ HS^−^ + OH^−^ (2)

HS^−^ + H_2_O ↔ H_2_S + OH^−^ (3)

(4) $$NH_{4}^{+}\;+\;H_{2}O\;↔\;NH_{3}\;+\;H_{3}O^{+}$$

H_3_O^+^ + OH^−^ → 2H_2_O (5)

Depending on the solution composition, the reactive agents include S^2−^, HS^−^ ions, and H_2_S molecules. Each of these reactive agents plays a distinct role in the sulfidation process. The less alkaline solution containing H_3_O^+^ appears to be more effective in attacking surface oxides [48]. In this context, the choice of solvent significantly affects the sulfidation, affecting both the reactivity of sulfur species and the removal of surface contaminants. The solvent not only facilitates the distribution of reactive sulfur species but also interacts with the semiconductor surface to enhance or inhibit the cleaning process.

The simplified schematic of the transformation of (NH_4_)_2_S components can be described as follows:

(6) $$2{NH}_{4}^{+}+S^{2-}\;↔\;{\mathrm{NH}}_{3}+{NH}_{4}^{+}\;+\;HS^{-}\;↔\;2{\mathrm{NH}}_{3}\;+\;H_{2}S$$

Using t-butanol with a lower dielectric constant shifts the equilibrium to the right [20]. However, it appears that the alkyl group of the solvent does not participate as a reagent during substrate treatment with (NH_4_)_2_S-based solution. It indicates that the solvent’s primary role is to affect the solution dynamics rather than directly chemically interacting with the substrate.

The formation of gas phases such as H_2_S or NH_3_ makes the (NH_4_)_2_S-based solution less predictable as an etchant. Therefore, such a solution has a higher potential for cleaning rather than passivating or removing semiconductor material layer by layer. The cleaning effect is primarily due to the interaction of sulfur with weakly bonded atoms on the surface and impurity elements. Diluted solutions and short cleaning times are effective in removing contaminants without deeply breaking A^III^-B^V^ chemical bonds. From this perspective, isopropanol as an alcohol solvent provides a more stable cleaning composition over time, resulting in a stronger cleaning effect with consistent results.

In line with the obtained data, additional treatment with a sulfur-based solution clearly improves surface morphology and reduces contact resistivity. This reduction may be attributed to the decrease in electroactive surface defect states. As mentioned in [3], etching in a solvent with a high dielectric constant enhances the adsorption of sulfur film on the substrate. This suggests that, for cleaning purposes, the influence of solvent should be minimized. However, the presence of the solvent promotes surface smoothing and determines the etching process and the attack of weak bonds by oxygen species.

Furthermore, the increase in the density of high-current areas correlates with a decrease in contact resistivity, indicating a local improvement in charge-carrier transport. The low surface roughness achieved through sulfur treatment minimizes roughness-induced current leakage, enhancing overall device performance. Another factor could be the enhancement of hole concentration in the near-surface layer due to the sulfur cleaning effect. Moreover, sulfur treatment may eliminate hydrogen segregation by removing semiconductor surface defects, which are preferred sites for hydrogen atom bonding and accumulation [16,49]. During contact annealing in an N_2_ + O_2_ + H_2_O atmosphere, hydrogen is released, contributing to the removal of surface defects and improving contact quality [45].

Summarizing the obtained results, sulfur treatment led to a smoother interface, reducing activation centers for preventing undesirable metal-semiconductor interactions. However, it should be taken into account that the smoother morphology and reduced availability of species for metal incorporation likely contribute to weaker linking. From the perspective of laser diode processing, using (NH_4_)_2_S cleaning enhances device performance and lifetime by minimizing defects and improving the reliability of the metal-semiconductor contact.

### 3.3. Microstructure of Annealed Contacts

**Figure 4:**
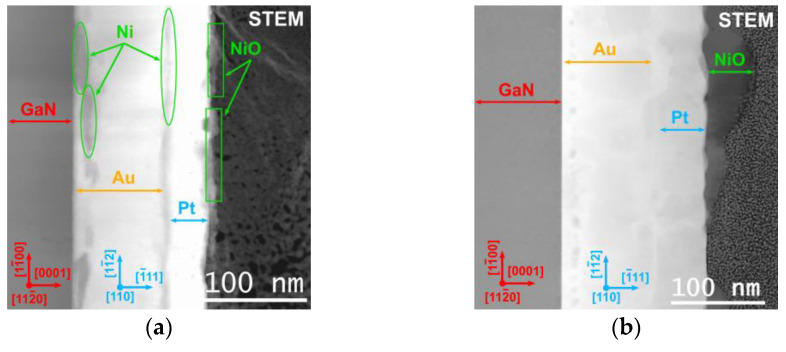
STEM image of annealed p-GaN/Ni/Au/Pt, after standard preparation (**a**) and with additional treatment by (NH_4_)_2_S solution (**b**).

To understand the mechanisms behind the reduction of specific contact resistance in the studied contacts on (NH_4_)_2_S-cleaned p-GaN substrates, we investigated the interfacial microstructure and contact morphological evolution using TEM and EDX analysis. Figure 4 compares the annealed contact microstructures of a standard prepared sample (Figure 4a) and a sample treated with (NH_4_)_2_S solution (Figure 4b). In both cases, the original sequence of the metal layers was altered after annealing. The alloyed contact film has a disordered solid solution structure. The standard-prepared sample exhibits a more compact arrangement of metallization, with a thickness of approximately 120–130 nm. In contrast, the (NH_4_)_2_S-treated sample shows a slightly thicker metallization layer, measuring about 150 nm.

TEM investigations did not reveal any intermediate semiconductor layer at the GaN/metal interfaces in samples additionally treated with the (NH_4_)_2_S cleaning solution. Metal species exhibited the same crystallographic orientation of [111] with respect to the [0001] GaN substrate (see Supplementary Materials Figure S1).

A line consisting of dark spots was observed about 10–12 nm away from the p-GaN/ Ni/Au/Pt interface additionally treated with (NH_4_)_2_S (Figure 4b). We associate these dark spots with Kirkendall voids rather than with Ni inclusions, since EDX data do not reveal an increased Ni signal at that region (see below). A similar effect of metal migration and voids was observed for Ni/Pt/Au contact, as described in [50]. This can also explain the slightly thicker metallization layer for the (NH_4_)_2_S-treated sample.

Upon annealing, the STEM image of the p-GaN/Ni/Au/Pt contact reveals a rough and non-uniform morphology. The thermal expansion differences among the metal components (which is [ppm/°F]: 7.2—for Ni, 7.9—for Au and 5.0—for Pt) as a source of mechanical stress allow them to become mobile, leading to inter-diffusion [51]. The metallization consists of a mixture of metal species with non-uniform propagation of Au in the vicinity of the interface, forming a sandwich structure with a Pt-rich layer and inclusions of indiffused gold and nickel in the middle. This mixing of metal species does not result in the formation of new alloys.

Most of the Ni diffuses across the gold and platinum layers. Upon reaching the surface, Ni reacts with oxygen molecules from the air, resulting in the formation of discontinuous NiO segregations randomly distributed on the surface. The high propensity of Ni species to oxidize, even in the presence of residual oxygen, is well-documented [13].

The reorganization of the contact composition could also induce additional stress, causing deformation of the initial metallization state. However, the boundaries between metal species after annealing remain clearly visible. According to [52], the out-diffusion of Ni species can lead to the removal of contamination layers from the semiconductor surface, thereby reducing the specific contact resistance. This may contribute to the lower *ρ_c_* observed in samples additionally treated with the (NH_4_)_2_S solution. Furthermore, the cleaning effect of the (NH_4_)_2_S solution may facilitate the easier dissociation of Mg complexes during annealing. As a result, the reactivation of acceptors in the near-surface layers of the semiconductor occurs [45,53].

In summary, the reorganization mechanism of the annealed metallization involves reshaping of morphology, mixing of metals, and oxidation of chemically unstable nickel. Increased thickness and transformed metallization morphology of the sulfur-treated substrate likely contribute to the reduction in specific contact resistance by providing a smoother interface and reducing activation centers for undesirable interactions.

**Figure 5:**
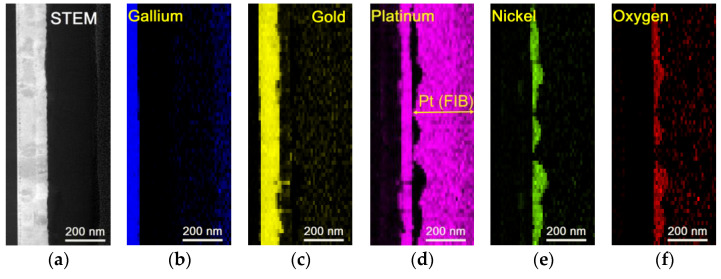
STEM image (**a**) and EDX maps of the element propagation (**b**–**f**) for the annealed p-GaN/Ni/Au/Pt system with treatment in (NH_4_)_2_S-based solution.

Figure 5a–d depict EDX maps of the Ni/Au/Pt contact on (NH_4_)_2_S-cleaned p-GaN substrate after annealing in an ambient atmosphere of N_2_ + O_2_ + H_2_O at 500 °C.

The inversion of all metal layers after annealing is evident. Random out-diffusion of Ni onto the top of the metallization results in islands of gold species matching with p-GaN and the rearrangement of the metallization during annealing. Annealing in an oxygen-containing environment leads to the oxidation of Ni, resulting in the formation of stable NiO on the platinum layer. Some gold also diffuses through the Pt layer to the top of the metallization. This suggests that the thermal energy during annealing is sufficient to drive the inter-diffusion of metal species. Further gold diffusion onto the surface is predominantly determined by NiO species. Despite the lack of metal reaction with the substrate, a Pt-rich layer contains inclusions of Au and Ni. EDX data confirm the absence of intermetallic compounds in the platinum layer containing Ni and Au.

Moreover, an intermediate semiconductor layer with a high p-type doping density and/or a low energy barrier was not detected between the metals and the p-GaN. No direct reaction between the metals and the p-GaN substrate indicates that the (NH_4_)_2_S cleaning is effective for preventing undesirable chemical interactions during annealing.

The EDX maps also reveal a good match between gallium and gold species at the interface. However, the presence of Au species near GaN does not result in gallium out-diffusion into the upper gold layer or the formation of cavities at the interface, as reported in [37,54]. The line of dark spots segregated in the Au layer probably corresponds to Kirkendall voids that migrate from the interface during annealing. This effect could be eliminated by an improved heat treatment procedure.

Nevertheless, obtained data confirm the positive influence of additional treatment with (NH_4_)_2_S-based mixture on the interface and the adhesion improvement of species. Formation of a clean and stable interface of p-GaN/Ni/Au/Pt samples additionally treated with (NH_4_)_2_S-based mixture may contribute to the electrical improvement of fabricated LDs.

**Figure 6:**
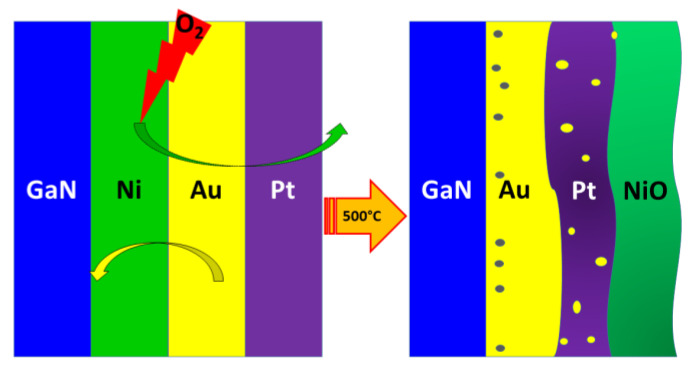
Schematic view on evolution of the annealed p-GaN/Ni/Au/Pt system with treatment in (NH_4_)_2_S-based solution (grey circles represent the voids).

After annealing samples additionally treated with (NH_4_)_2_S-based solution, the following changes are observed (Figure 6):

1. Morphological reorganization of the contact system.
2. Formation of a more complex metallization structure.
3. Complete oxidation of nickel into NiO.
4. Extensive diffusion of metal species leading to the formation of the Pt-Au/Ni solid mixture.
5. Absence of a heterostructural intermediate semiconductor layer at the GaN/metallization interface.
6. Lack of the chemical reaction between the metals and the p-GaN substrate.
7. Presumably, void generation in the Au layer and migration during annealing.

**Figure 7:**
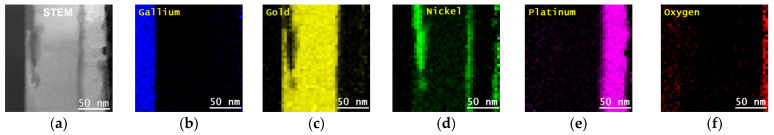
STEM image (**a**) and EDX maps of the element dispersion (**b**–**f**) for the annealed p-GaN/Ni/Au/Pt system after standard cleaning.

Figure 7 presents STEM and EDX data of standard-prepared samples after annealing. A somewhat blurred GaN/metal interface on the EDX map reveals the formation of GaN-Au inter-diffusion layer with a thickness of about 3 nm at the interface after the annealing process. The formed interlayer disrupts the initial carrier balance, potentially leading to higher contact resistivity due to the altered electronic properties at the interface. Along with the method of metal de-processing, the EDX maps confirm stronger bonding between the semiconductor and metallization after annealing, evidenced by the formation of a well-defined inter-diffusion layer. However, migration of metal species is less pronounced compared to samples additionally cleaned with (NH_4_)_2_S-based solution. Heated gold has a loosely packed structure and creates a broader mixing layer than initially. As in case of annealed p-GaN/Ni/Pt/Au contact system, described in [50], the thickness of separated metal layers has no influence on gold migration to the GaN surface.

Incorporated pure Ni islands are mainly separated from the interface by a thin layer of gold. Additionally, Ni forms a solid solution with Au in the middle of metallization, suggesting a complex diffusion process during annealing. Perhaps Ni accumulation in the form of an island in the gold layer occurs due to stress-induced inter-diffusion and time limits for full out-diffusion onto the surface [55].

The increased separation of metal species, such as Ni and its oxidation, accommodates more misfit surface dislocations in the annealed metallization system [56]. This could lead to localized strain and affect the overall stability of the contact. Similar compositions have been observed by other researchers as well [13]. The most uniform layer of nickel is situated at the GaN-Au and Pt-Au boundaries, indicating slower diffusion of metals and reduced out-diffusion and oxidation of nickel. The preferential diffusion of metal atoms likely occurs within dislocations, providing pathways for faster diffusion. This behavior is crucial for understanding how annealing affects the distribution and interaction of metal species within the contact structure.

In samples with standard cleaning, the metal migration is more pronounced, leading to a less stable and uniform interface. This additionally highlights the effectiveness of sulfur-based cleaning in maintaining interface integrity during annealing.

**Figure 8:**
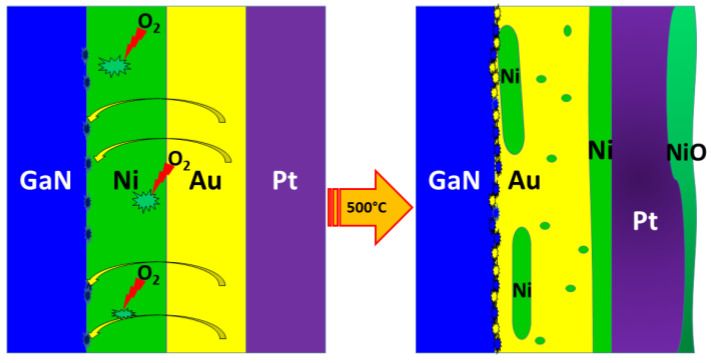
Schematic view on evolution of the annealed p-GaN/Ni/Au/Pt system after standard cleaning.

In the case of standard-prepared samples after annealing, several significant changes were observed (Figure 8):

- Morphological and compositional transformation of the contact system.
- Formation of a GaN-Au intermediate layer at the interface (see Supplementary Materials Figure S2).
- Uneven diffusion of metal species.
- Formation of an Au-Ni solid solution with inclusions of pure Ni islands.
- Fractional oxidation of nickel into oxide, revealing a multilayer structure.

In both treatment procedures, the initial intermediate nickel layer did not prevent gold from reaching the metal-semiconductor interface, nor did the platinum layer hinder the metallization oxidation. This suggests that neither layer acts as an effective barrier against diffusion and the oxidation process during annealing.

Unlike findings in [57], there was no evidence of lattice mismatch influencing the subsequent behavior of the metals during annealing. This implies that the inter-diffusion observed is primarily due to thermal effects rather than lattice compatibility issues.

Standard-prepared samples exhibited greater inter-diffusion of metal species, which can introduce more scattering points and increase contact resistivity. The more pronounced intermixing and formation of scattering centers led to higher electrical resistance at the metal-semiconductor interface. On the other hand, additional treatment with (NH_4_)_2_S prevents complete metal mixing and promotes the formation of Kirkendall planes and voids, which were not observed in samples prepared using standard methods.

The oxidation process typically roughens the metal surface significantly and alters its initial texture. In both cases, Ni migration and oxidation led to the formation of hillock-like structures on the surface. No repeatable pattern was observed. Irregularly sized and shaped hillocks are more prominent in samples treated with (NH_4_)_2_S solution due to the higher amount of NiO present on the surface. Otherwise, complete oxidation of Ni may eliminate further diffusion of metals and therefore contribute to the more stable electrical parameters of contacts.

In summary, the reorganization mechanism of the annealed metallization involves reshaping the morphology, mixing of metals, and oxidation of chemically unstable nickel. The increased thickness and transformed metallization morphology of the sulfur-treated substrate likely contribute to the reduction in specific contact resistance by providing a smoother interface and reducing activation centers for undesirable interactions that could degrade the electrical parameters of contact over time.

From the perspective of laser diode processing, we prioritize the cleaning effect over passivation, which tends to be less predictable. Prolonged treatment can result in a non-uniform, island-like formation of an amorphous sulfur film on the semiconductor surface [29]. These less controllable and reproducible outcomes from extended treatment prevent us from clearly identifying its influence on specific contact resistance.

The sulfur film can effectively act as a barrier during annealing, preventing metal diffusion into GaN [29]. However, it is important to note that in the case of epitaxial-quality substrate surfaces, the deposited sulfur tends to bond weakly through chemisorption [31,58]. Stronger chemical bonds can be achieved through prior surface preparation or the creation of dangling bonds on the semiconductor surface. Moreover, additional treatment is required to create a more homogeneous and stable sulfur film, which would alter the overall process.

In the context of LD fabrication, however, a sulfur film is undesirable. It weakens the bonding between the semiconductor and the metallization layer. For sulfur deposition, the wet treatment with (NH_4_)_2_S-based solutions should follow with the additional modifications of passivation film or the epitaxial semiconductor surface before the etching process.

Our experiments did not reveal a significant difference in the operating voltage of fabricated laser diodes between standard cleaning and additional treatment with (NH_4_)_2_S solution. Statistical data (see Supplementary Materials Figure S3a,b) indicate that additional treatment with (NH_4_)_2_S solution results in a more stable contact system. The negligible difference in operating voltage between laser diodes fabricated using standard cleaning and those with additional (NH_4_)_2_S treatment could be attributed to the mixed nature of the metallization. These findings suggest that the short-duration (NH_4_)_2_S treatment primarily influences the contact system by cleaning and smoothing the semiconductor surface and limiting semiconductor-metal reactions rather than affecting metallization behavior during annealing.

From the perspective of laser diode processing, treatment with (NH_4_)_2_S solution appears to be primarily useful for its cleaning effects rather than enhancing the electrical properties significantly. This cleaning procedure has potential from the point of view of saving the initial GaN epitaxial layer and further device reliability.

Additionally, we do not recommend etching with (NH_4_)_2_S to achieve the sulfur film. The weak stability of the solution complicates consistent substrate preparation, making it less reliable for achieving reproducible results. Passivation effect is effective for semiconductor-metal separation. However, the amorphous nature of deposited sulfur may reduce the adhesion of the metal structure to the semiconductor wafer. The requirement for metallization annealing during LD fabrication determines sulfidation of the semiconductor surface due to breaking bonds at the time of heating at temperatures higher than 300–400 °C [20]. This is critical for ensuring proper adhesion and electrical contact. Furthermore, improvements in electronic properties are temporary due to the instability of the passivated layer [47]. This point is especially important during the last stage of laser diode fabrication, where consistent performance is essential.

One challenge in integrating (NH_4_)_2_S treatment into existing LD fabrication processes is the potential degradation of metal composition without appropriate protection during processing. The effects of cleaning, smoothing, or passivation with a sulfur film should be carefully controlled by adjusting treatment parameters depending on the specific goals of the fabrication process. For LDs, we recommend a short treatment duration to avoid sulfur deposition without proper film stabilization.

Additionally, incorporating (NH_4_)_2_S treatment into the fabrication workflow may pose difficulties, particularly in terms of sequencing the processing steps and maintaining the stability of the initial surface state (in the case of covering with the sulfur film).

The obtained research results underscore the need for meticulous control over surface treatments and annealing conditions in semiconductor device fabrication, particularly for laser diodes. While (NH_4_)_2_S treatment offers effective cleaning, its impact on long-term electronic properties and adhesion must be carefully managed. Achieving stable and low-resistance contacts requires a comprehensive understanding of metal diffusion and oxidation behavior, as well as the development of robust and consistent processing techniques.

## 4. Conclusions

The (NH_4_)_2_S-solvent cleaning process, combined with subsequent metal inversion and nickel oxidation, potentially eliminates weak bonds on the GaN surface, removes contamination layers from the interface, keeps the initial state of the GaN epitaxial layer, and decreases specific contact resistance. The (NH_4_)_2_S-isopropanol solution provides the most superior performance in terms of reducing specific contact resistance (up to *ρ_c_* < 1 × 10^−4^ Om·cm^2^) and smoothing the substrate surface (up to *R_a_* = 4 nm).

The heat treatment results in the intimate contact formation between GaN and metallization, which is particularly evident on the sulfur-treated semiconductor surface, and facilitates metal diffusion within semiconductor dislocations in standard-cleaned samples. Annealing of the contact system in an N_2_ + O_2_ + H_2_O atmosphere at 500 °C caused degradation through species inter-diffusion and metal-metal solid solution formation, irrespective of the preparation method. In both cases, the metal migration and Ni oxidation caused the surface modification and formation of irregularly sized and shaped hillocks, which was more obvious for samples prepared by (NH_4_)_2_S solution due to the higher amount of NiO on the surface.

The (NH_4_)_2_S-solvent cleaning eliminates GaN decomposition and complete metal mixing during contact system annealing. It was established that the additional treatment with sulfur solution shows no significant influence on the chemical reaction of nickel species prone to oxidation but provides less mixing of metals in comparison with the standard preparation process.

The research results suggest that incorporating an (NH_4_)_2_S-based solution into the cleaning protocol could be a valuable step in the fabrication process of GaN-based LDs. However, it can be used for short-term treatment to obtain the cleaning and smoothing effect. Decreasing surface roughness after treatment with (NH_4_)_2_S-based solution has a stronger influence for a sharper/more imperfect initial surface. Longer treatment causes passivation, which, in the case of LD fabrication, may provide weaker bonding between the semiconductor and metals.

This treatment procedure may lead to higher yield and reliability in optoelectronic device manufacturing. However, the conditions of treatment with (NH_4_)_2_S-based solution should be applied in line with appropriate parameters of the devices and be specific to the fabrication process.

## Figures and Tables

**Table 1:** AFM data of surface roughness and the specific contact resistance of p-GaN/Ni/Au/Pt.

Etching Composition	Surface Roughness, *R_a_* [nm]	Specific Contact Resistance, *ρ_c_* [×10^−4^ Ω·cm^2^]
(NH_4_)_2_S-t-(CH_3_)_3_COH	9.0	0.7
(NH_4_)_2_S-(CH_3_)_2_CHOH	4.0	0.1
(NH_4_)_2_S	12.0	0.4
Standard cleaning	16.0	3.3

## Data Availability

The data are contained within the article or Supplementary Materials. The data presented in this study are available on request from the corresponding author. The data are not publicly available due to privacy reasons.

## Supplementary Materials

The following supporting information can be downloaded at: https://www.mdpi.com/article/10.3390/ma17184520/s1, Figure S1: STEM image of annealed p-GaN/Ni/Au/Pt with additional treatment by (NH_4_)_2_S solution (a), and the crystallographic orientation (b) determined by FFT. ZA—zone axis.; Figure S2: HR-TEM image of the interface of the annealed p-GaN/Ni/Au/Pt after standard preparation. Figure S3: Evolution of the operational voltage of the annealed p-GaN/Ni/Au/Pt contact system with standard and (NH_4_)_2_S preparation: with (a) time; (b) statistical changes.
