# Supplementary material for: Pros and Cons of (NH4)2S Solution Treatment of p-GaN/Metallization Interface: Perspectives for Laser Diode
Source: Materials (Basel). 2024 Sep 14;17(18):4520. doi: 10.3390/ma17184520 (PMC11433334; doi:10.3390/ma17184520)
Supplement: Supplementary file 1 [file materials-17-04520-s001.zip › materials-3171994-supplementary.pdf]

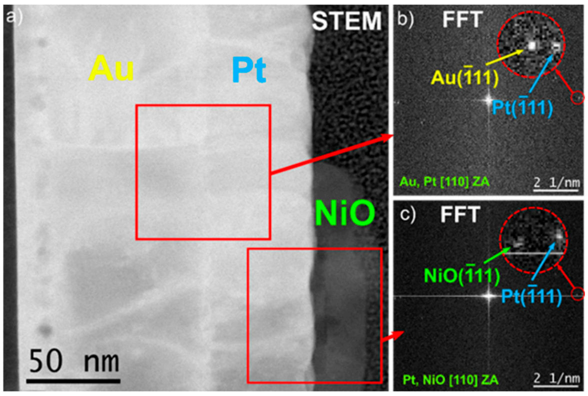

Figure S1. STEM image of annealed p-GaN/Ni/Au/Pt with additional treatment by (NH4)2S solution (a), and the crystallographic orientation (b) determined by FFT. ZA – zone axis.

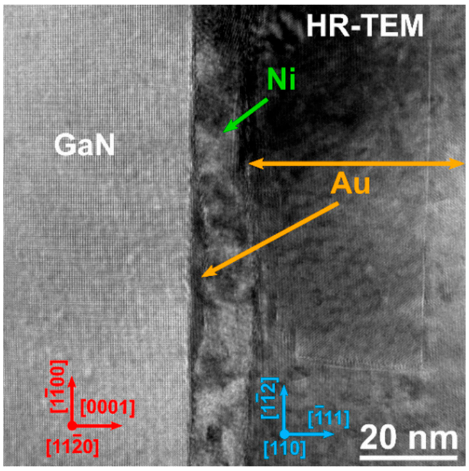

Figure S2. HR-TEM image of the interface of the annealed p-GaN/Ni/Au/Pt after standard preparation.

Stability of annealed contact.

Lasers with both standard and $(NH_4)_2S$ treatment were mounted in TO-56 housings. Lifetime measurements were conducted at a stable temperature of 25°C and a current of 80 mA over 150 hours to assess changes in the operating voltage.

The comparison of operating voltage evolution at 80 mA over the 150-hour period is shown in Figure S3a. Compared to the $(NH_4)_2S$-treated lasers, the standard-treated devices exhibited a larger change in operating voltage and slower stabilization. However, in both cases, slight degradation of the contact system was observed. This trend may be attributed to the contact system itself and warrants further improvement and investigation.

Figure S3b represents an average statistic for multiple lasers, showing that the $(NH_4)_2S$-treated devices have less variation in voltage change and smaller overall values.

Based on these results, additional treatment with $(NH_4)_2S$ appears beneficial for improving ohmic contact stability and enhancing the lifetime of laser diodes.

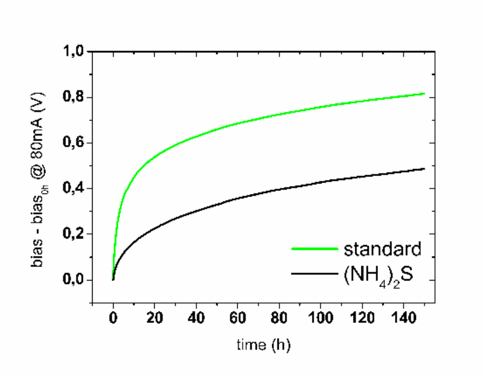

(a)

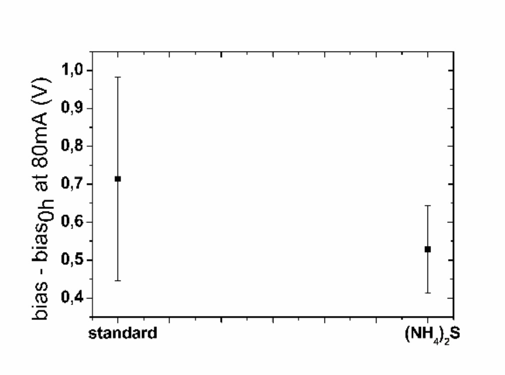

(b)

Figure S3. Evolution of the operational voltage of the annealed p-GaN/Ni/Au/Pt contact system with standard and $(NH_4)_2S$ preparation: with a) time; b) statistical changes.
